# The antiangiogenic agent ZD4190 prevents tumour outgrowth in a model of minimal residual carcinoma in deep tissues

**DOI:** 10.1038/sj.bjc.6605092

**Published:** 2009-07-21

**Authors:** K Gaballah, R Oakley, A Hills, A Ryan, M Partridge

**Affiliations:** 1 Department of Oral and Maxillofacial Surgery, King's College London, Guy's King's and St Thomas’ Hospitals, Guy's Tower, Great Maze Pond, London SE1 9RT, UK; 2 Department of Cancer Biosciences, Astrazeneca, Alderley Park, Macclesfield, Cheshire SK10 4TF, UK

**Keywords:** minimal residual carcinoma, antiangiogenesis, head and neck, therapy

## Abstract

**Background:**

Tumour cells may persist at the operative site after seemingly adequate surgery. Radiotherapy is often given in an attempt to prevent repopulation, but this modality cannot be relied upon to prevent locoregional recurrence. An alternative strategy is to take advantage of the requirement of tumour cells to develop an independent blood supply and block this process to prevent recurrence.

**Methods:**

In this study, we evaluate the effect of the angiogenesis inhibitor, ZD4190, using a rodent model of residual carcinoma in deep tissues, mimicking the clinical scenario where low numbers of malignant cells persist at the operative site.

**Results:**

The tumour burden that could be eliminated was dependent on the site where the cells were implanted. Immediate treatment with ZD4190 prevented outgrowth of up to 2.5 × 10^5^ cells in the rectus muscle and 1 × 10^5^ in the gastrocnemius, whereas control animals developed large tumours. When more than 2.5 × 10^6^ cells were implanted into the rectus or 1 × 10^6^ into the gastrocnemius and treatment was maintained for 3 weeks, the carcinomas that developed in ZD4190-treated animals showed a reduced microvessel density and increased necrosis when compared with the vehicle-treated controls, but an infiltrative growth pattern was common.

**Conclusion:**

These findings suggest that antiangiogenic agents have a role to play in preventing outgrowth of residual carcinoma and are likely to be most effective when the tumour burden is minimal.

The high rate of recurrence after seemingly adequate surgery and radiotherapy for tumours arising in the head neck area implies the presence of residual disease. Studies using molecular diagnostics, based on finding the same *p53* mutation in a carcinoma and the surrounding normal tissues, have confirmed the presence of malignant cells in tissues assessed as being tumour free by the pathologist (Brennan *et al*, 1995; Partridge *et al*, 2000; van Houten *et al*, 2002, 2004). Most effort has been focused on detection of residual carcinoma at the operative site as locoregional recurrence, rather than distant metastasis, is the most important cause of treatment failure for these cases (Partridge *et al*, 2005).

The samples that are forwarded for this ultrasensitive analysis are referred to as ‘molecular margins’. This is important as the term surgical margins frequently causes confusion, as it may be wrongly assumed that the tissues examined are taken from the edge of the resected carcinoma, rather than from the normal tissues. Analysis of both mucosal and deep ‘molecular margins’ has revealed that the finding of p53 mutations in deep margins is more strongly associated with development of local recurrence than *p53*-mutation-positive mucosal margins (Huang *et al*, 2007). A second key observation that has emerged from all studies applying these new diagnostics (Brennan *et al*, 1995; Partridge *et al*, 2000, 2005; van Houten *et al*, 2002, 2004; Huang *et al*, 2007) is that multiple tumour-positive margins are associated with local recurrence, indicating that local spread is more widespread than envisaged earlier. As the prognostic significance of this residual carcinoma becomes clearer, the challenge is to find new ways of preventing outgrowth of these remaining malignant cells. At present, patients with close margins after surgery, two or more tumour-positive lymph nodes or histological features that suggest aggressive disease receive postoperative radiotherapy, yet more than half of these cases develop locoregional recurrence. The presence of a *p53* mutation in these cancers may be relevant but is only one of the many factors that influence the response to radiotherapy such that new treatments need to be added to conventional protocols if outcome is to be improved.

It is well established that tumours induce changes in the vasculature and extracellular matrix and that malignant cells must develop an independent blood supply to grow beyond a critical size. In this study, we evaluated the ability of ZD4190, an orally available inhibitor of the vascular endothelial cell growth factor receptor 2 (VEGFR2) and of epidermal growth factor receptor (EGFR) signalling, to block the development of vasculature required to support outgrowth of tumour cells (Wedge *et al*, 2000; Ryan and Wedge, 2005). Vascular endothelial cell growth factor plays a pivotal role in endothelial cell mitogenesis and chemotaxis, modulating cellular–extracellular matrix interactions (Wedge *et al*, 2000). These activities facilitate vessel sprouting and capillary tube formation. Vascular endothelial cell growth factor may also contribute to tumour progression through its permeabilising effect on the vasculature (Dvorak *et al*, 1995; Roberts and Palade, 1997), and activation of VEGFR2 appears sufficient to promote the major phenotypic responses to VEGF, including endothelial cell proliferation, migration, survival and the induction of vascular permeability (Wedge *et al*, 2002).

We used a preclinical model where defined numbers of cells are implanted into different muscles, mimicking the clinical scenario where residual tumour persists in deep structures postoperatively (Oakley *et al*, 2002). We selected an immunocompetent host and a cell line that forms infiltrative tumour tracts in muscle, rather than a subcutaneous model, as most recurrence occurs in deep tissues and the local delivery of novel therapeutics to this site is not straightforward (Oakley *et al*, 2002). The cell line also carries a *p53* gene mutation. This is of clinical relevance when evaluating antiangiogenic agents as tumours lacking *p53* may show reduced apoptosis and a reduced treatment response under hypoxic conditions (Yu *et al*, 2002). In pilot studies, we considered the feasibility of developing a model of residual tumour by resecting the greater portion of an established carcinoma. However, the amount of residual tumour was unpredictable, whereas implanting defined numbers of malignant cells provided a reproducible way to examine the effects of an antiangiogenic agent at different time points as the tumour foci developed an independent vasculature.

## Materials and methods

### Establishment of the model of minimal residual cancer

Female mice were obtained from Harlan Ltd (Oxford, UK) and were used in accordance with institution guidelines when they were 7–8 weeks old. Two tracts of malignant squamous keratinocyte PDVC57B cells, which are syngeneic for C57Bl/6 mice and mimic the clinical problem by invading into muscle, were implanted under halothane anaesthesia using a 30-gauge needle, into each gastrocnemius muscle and five tracts were implanted into the larger rectus muscle. This cell line carries a p53 mutation at codon 231 (ATG-CTC) and an A–T transversion at codon 61 of the H-ras gene (Oakley *et al*, 2002). A range of cells was implanted (1–5 × 10^4^–5 × 10^5^ per tract), injecting as the needle was withdrawn (Oakley *et al*, 2002). In some experiments, the tumour was resected under anaesthesia, aiming to leave residual carcinoma, and the wound closed with sutures.

### Drug treatment

ZD4190 was administered by gavage suspended in 1% polysorbate 80 at 50 mg kg^−1^ 24 h after tumour cell implantation for a maximum of 22 days. Control rodents were gavaged with the vehicle only and a third group was untreated. In preliminary experiments, rodents were killed on alternate days so that the effect of ZD4190 on tumour outgrowth could be assessed and subsequently the treatment response was evaluated at day 9 or 22. At least five rodents were included in each treatment group.

### Histopathological assessment

At appropriate time points, the animals were killed and the tumour-bearing muscles removed for histological examination. Tissues were fixed in zinc buffer (0.5 g calcium acetate, 5 g zinc acetate, 5 g zinc chloride dissolved in 1 l of 0.1 M Tris, pH 7.4) for 8–12 h. Each muscle was bisected at the midpoint and the two halves embedded, with the cut surface uppermost, and processed into wax. General tissue morphology was evaluated by the examination of haematoxylin- and eosin-stained sections. The combined area of the tumour tracts and any areas of necrosis were measured using a Leica Q500IW workstation (Oakley *et al*, 2002).

### Establishing the time point at which neoangiogenesis occurs

To determine when proliferating endothelial cells were present in the developing tumour tracts, pairs of rodents implanted with tumour cells received bromodeoxyuridine (BrdU) at 40 mg kg^−1^ 40 min before killing on days 4, 6, 8, 10, 14, 16 and 18. Tissues were processed as described above and step-serial sections examined at 100-*μ*m intervals.

### Immunohistochemistry

Pilot studies with antibodies recognising CD31, CD34, VWF and CD105 (all BD Biosciences, Abcam, Cambridge, UK) identified rat anti-mouse CD31, diluted 1 : 20 as the most appropriate reagent to estimate the number of blood vessels present in the tumour tracts. The secondary antibody was donkey anti-rat alkaline phosphatase (Jackson ImmunoResearch, Stratech, Soham, UK) diluted 1 : 200 followed by development with the alkaline phosphatase substrate kit (Vector Laboratories, Peterborough, UK). Sections incubated with antibody dilutant were used as negative controls. Immunolocalisation of VEGF was with goat anti-mouse VEGF (1 : 200; Sigma, Poole, Dorset, UK), of VEGFR2 was with rabbit anti-VEGFR2 (1 : 100; Zymed, Invitrogen, Paisley, UK) and of cytokeratins with guinea pig anti-mouse (1 : 100; Progen, Heidelberg, Germany).

Step-serial sections were examined by double labelling for CD31 and BrdU. After staining for CD31, BrdU was detected with the *in situ* detection kit (BD Biosciences, Oxford, UK) with biotinylated anti-BrdU (1 : 10 for 1 h followed by development with streptavidin–horseradish peroxidase for 30 min and visualisation with DAB (3,3-diaminobenzidine; DAKO, Ely, UK)). Proliferating endothelial cells were scored as such when they expressed CD31 and BrdU and were associated with tubular structures. The percentage of double-stained cells was estimated by counting 200 nuclei. We did not find any evidence that PDVC57B cells expressed CD31 or of cytokeratin-positive cells lining vessels containing red blood cells, suggesting that it is unlikely that vascular mimicry (Hendrix *et al*, 2000; Sood *et al*, 2001; Shieh *et al*, 2004) contributes significantly to the angiogenic response.

### Determination of microvessel density following implantation of tumour cells

Microvessels were counted within the tumour foci at the midpoint of each muscle at days 9 and 22. At the first time point, all tumour vessels were counted but at day 22, varying patterns of coalescing tracts were observed and the vessels were counted for four fields at × 100 magnification, selecting one within each quadrant of the tumour, avoiding any areas of necrosis or cystic cavities. The counts were expressed as the mean±s.e. for an area of 80 *μ*m^2^ as this was the cross-sectional area of the smallest focus of tumour measured at this time.

### Cytotoxicity assay in monolayer culture

The cytotoxicity of ZD4190 for PDVC57B cells was established when 10^4^ cells were exposed to 1–10 *μ*M ZD4190 for 96 h before MTS solution (Promega, Southampton, UK) was added and the optical density measured at 490 nm. Cells were also grown to 40% confluence and treated with 1–100 *μ*M ZD4190 for 7 days and the cytopathic effect examined by staining with crystal violet.

### Statistical analysis

One-way ANOVA was used to compare the microvessel density (MVD) and the combined area of the tumour tracts at the midpoint of the muscles for groups of rodents treated with ZD4190 and the vehicle-treated controls. SPSS 11.1 (SPSS.com, Woking, UK) for windows was used and a *P*<0.05 was considered statistically significant.

## Results

### Recapitulating outgrowth of residual carcinoma in deep tissues

**Figure 1:**
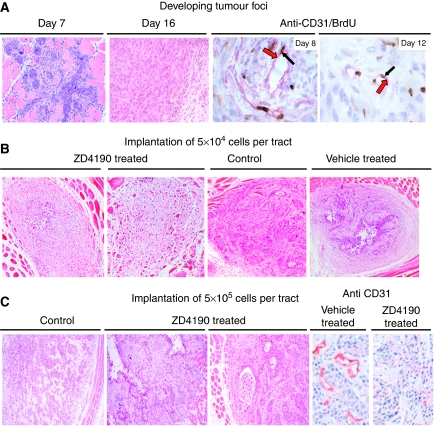
Effect of ZD4190 on tumour outgrowth in a model of residual cancer in the muscle, recapitulating the clinical scenario where malignant cells remain in the muscle after surgery. (**A**) Morphology of a representative tumour tract in the rectus muscle 7 and 16 days after implantation of PDVC57B cells; magnification: × 240 (left) and × 280 (right). Identification of proliferating endothelial cells by double staining for CD31 (red arrow) and BrdU (black arrow) at the tumour periphery at day 8 and core at day 12 (magnification, × 1000). (**B**) Gavage with ZD4190 prevented the outgrowth when up to 5 × 10^4^ PDVC57B cells were implanted to create each tumour tract. Two representative examples of the fibrotic foci found at day 22 are shown (far left, × 100) together with a fibrotic focus with viable malignant cells (centre left, × 100). In contrast, control rodents developed large tumours (centre right, × 100), as did those receiving vehicle alone although some lesions that developed in this group were associated with peripheral areas of fibrosis (far right, × 100). (**C**) When higher numbers of malignant cells were implanted to create each tract (>1 × 10^5^), large tumours developed in the control and vehicle-treated groups by day 22 ( × 80). The tumours present in the ZD4190-treated rodents typically showed a more infiltrative pattern of growth with cords of cells, areas of necrosis and reduced microvascularity, as determined by CD31 staining, when compared with those that developed in the vehicle-treated group (far right, × 240).

In pilot studies, the greater portion of an established carcinoma was resected to mimic the clinical scenario where tumour remains at the operative site, but the amount of residual carcinoma was unpredictable (data not shown). In contrast, implanting defined numbers of malignant cells provided a reproducible way to examine the effects of an antiangiogenic agent. When 1–5 × 10^5^ PDVC57B cells were implanted to create each tumour tract, proliferating malignant keratinocytes were detected after 96 h. The tumour foci included fibroblasts and inflammatory cells, and by day 7, cords of malignant cells extended between the muscle fibres (Figure 1A). Typically, the tumour had a well-circumscribed margin and large venules and arteries, and smaller vessels were apparent after 16 days as well (Figure 1A). Implanting 10^4^ cells to create each tract resulted in delayed tumour outgrowth, with cords of 2–4 layers of malignant cells being the most frequent finding. However, rapidly growing tumour foci developed when 2–5 × 10^4^ cells were implanted. The efficiency of tumour tract formation at day 9, for groups of 10 rodents implanted with the same number of cells, ranged from 76 to 95% when the results from three consecutive experiments were assessed.

### Detection of VEGF and VEGFR2 and proliferating endothelial cells by anti-CD31 and BrdU staining

Vascular endothelial cell growth factor and VEGFR2 were detected in clumps of PDVC57B cells and in the stroma at day 6. Following double staining for BrdU and CD31, proliferating endothelial cells were identified at the edge of the tumour, with vascular sprouts, chains of endothelial cell and hyperplastic vessels detected from day 8 (Figure 1A). Approximately 3% of the tumour endothelial cells were positive with both markers. Similar structures were present in the centre of the tumour at day 12 (Figure 1A), but the number of cells implanted did not influence the time when proliferating endothelial cells were detected.

### ZD4190 prevents outgrowth of residual tumour

When 2 × 10^4^ PDVC57B cells were implanted to create each tumour tract in the gastrocnemius (total of 4 × 10^4^ cells) and rectus (total of 1 × 10^5^ cells) muscles, no tumours were detected clinically for the rodents treated with ZD4190. Histological assessment of tissues at day 22 did not reveal any evidence of tumour in 16 of 20 muscles implanted with a total of 35 tracts, but rather fibrotic foci with collagen fibrils, fibroblasts and scattered inflammatory cells replaced the muscle (Figure 1B). CD31 staining showed that many of these fibrotic foci contained large vessels, and vascular hot spots were identified in the adjacent muscle. Four of the tumour-bearing muscles in the test group (three rectus and one gastrocnemius) contained fibrotic foci with central islands of proliferating tumour as determined by BrdU staining, but these treatment failures were confined to two rodents (Figure 1B). Viable carcinoma cells, which were not actively proliferating, were also detected within two fibrotic foci in the rectus (Table 1). Fibrotic foci were also the predominant finding at sites where 5 × 10^4^ cells were implanted to create each tumour tract (a total of 2.5 × 10^5^ cells for the rectus and 1 × 10^5^ cells for the gastrocnemius) and the rodents gavaged with ZD4190 (Table 1). Similar treatment effects were seen when these experiments were repeated with other groups of animals. In contrast, the tumours that developed in the control groups were highly cellular, with darkly stained nuclei (Figure 1B) although a proportion (10%) developing in the rectus, in the group gavaged with the vehicle only, was associated with peripheral fibrotic reactions that were not seen in the untreated group (Table 1, Figure 1B).

**Figure 2:**
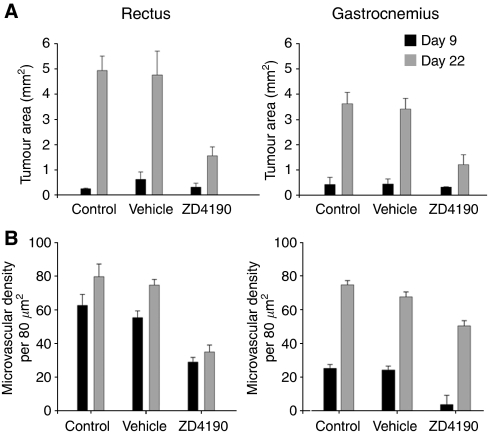
Treatment with ZD4190 reduced the area and microvascularity of tumour foci in a muscle model of minimal residual carcinoma. ZD4190 reduced (**A**) the tumour area at day 22 when more than 10^5^ PDVC57B cells were implanted to create each tract in the rectus and gastrocnemius muscles for groups of mice (*n*=5) and (**B**) the MVD of the tracts that developed in the gastrocnemius at day 9 and at both sites at day 22.

When more than 10^5^ cells were implanted to create each tract, fibrotic foci were rarely detected and the combined area of the tracts at the midpoint of each muscle was broadly similar (Figure 2A) although the total number of cells implanted into each muscle was different. The MVD of the tumours developing in the rectus was consistently higher than that observed for the gastrocnemius (Figure 2B), and following treatment with ZD4190, there was a reduction in the MVD for the tumours in the gastrocnemius at day 9 (*P*=0.00, Figure 2B) but not for those in the rectus.

By day 22, large tumours were apparent in all groups, with clumps and cords of squamous cells admixed with loose stroma. Many tumours had a well-circumscribed margin (Figure 1C, far left) but others showed an infiltrative growth pattern and areas of central necrosis. The tumours that developed in the ZD4190-treated group were characterised by a heterogeneous growth pattern, with dense islands of carcinoma and admixed stroma or cord-like proliferations of malignant cells (Figure 1C). Cystic spaces were located at the periphery in the ZD4190-treated group (Figure 1C). At this time, the tumour area was reduced in the rectus (Figure 2A, *P*=0.004) and the fraction of the tumour area showing necrosis was increased when ZD4190-treated (22.32%±4.79) and vehicle-treated groups (4.93%±3.17) were compared (*P*=0.00). A similar reduction in the tumour area, and a corresponding increase in the fraction of the tumour area showing necrosis, was found for the gastrocnemius (21.37%±3.65 *vs* 4.72%±2.61, *P*=0.001). A reduction in the MVD was apparent when the ZD4190- and vehicle-treated groups were compared (*P*<0.005, Figure 1C, far right, and Figure 2B) but these tumours showed a more infiltrative growth pattern when compared with those arising in the control group. In contrast to the inhibitory effect of ZD4190 on tumour outgrowth *in vivo*, no significant cytotoxicity was observed following exposure of PDVC57B cells to 0.1–100 *μ*M ZD4190 *in vitro*.

## Discussion

Translational research, based on the application of molecular diagnostics to detect residual tumour after surgery for head and neck carcinoma, has highlighted the need to identify treatment modalities, other than radiotherapy, to prevent recurrence at the operative site (Huang *et al*, 2007). Vascular endothelial cell growth factor signalling inhibitors are attractive for the treatment of residual disease, as tumour regrowth is dependent on neovascularisation, and in this report, we show that ZD4190 can prevent outgrowth of malignant cells when the tumour burden is below a critical threshold.

We developed preclinical models implanting varying numbers of malignant cells to mimic the clinical situation where residual carcinoma interacts with the surrounding host cells and matrix to obtain nutrients and subsequently to develop an independent vasculature. Different numbers of cells were implanted so that the effect of ZD4190 could be tested against different tumour burdens. We identified 2 × 10^4^ cells per tract as the lowest number that reproducibly generated viable tumour tracts and implanted a maximum of 2.5 × 10^6^ cells when considering the treatment effect with a higher tumour burden. The onset of endothelial cell proliferation was established by dual immunostaining as reagents that differentiate between new and established tumour vessels, for example the murine homologues of magic roundabout (Huminiecki *et al*, 2002) or tumour endothelial cell markers (Nanda *et al*, 2004a,2004b), are not yet available. Proliferating endothelial cells were detected at the tumour periphery at day 8 and at the centre from day 12. However, double-stained cells were difficult to detect and our step-serial approach must have missed these events, at least in the rectus, as the MVD for this muscle was greater than that for the gastrocnemius at day 9.

We anticipated that implanting different numbers of PDVC57B cells would result in differences in the area of the tumour tracts at the early time point, but no such relationship was found. However, when five tracts, each with 1 × 10^5^ cells, were implanted into the rectus, the MVD was consistently higher than that when two tracts (2 × 10^5^ cells in total) were introduced into the gastrocnemius. The different microvessel densities are explained on the basis that MVD reflects the balance of pro- and antiangiogenic stimulators in the tissue environment and is not only a measure of the metabolic burden or the angiogenic dependence of the supported tumour cells (Hlatky *et al*, 2002). The higher MVD score for the carcinomas developing in the rectus indicates that implanting more malignant cells into this muscle provides a greater angiogenic signal than that when fewer cells are implanted into the gastrocnemius. However, any new vessels are unlikely to be functional at day 9, as the tumour area is similar for both muscles, indicating that proliferation is limited by the available nutrients.

We started treatment immediately after tumour cell implantation, because when treating patients it makes sense to give adjuvants early, as the growth of residual malignant cells may be stimulated when the wound bed contains many growth factors. We showed that following implantation of small numbers of malignant cells (a maximum of 5 × 10^4^ cells per tract), ZD4190 has the potential to prevent tumour outgrowth. The most frequent findings were fibrotic foci that represent a spectrum of change as hypoxic malignant cells die elicit an inflammatory response, phagocytes clear debris and granulation tissue is remodelled. Typically, these were associated with vascular hot spots and this enhanced blood supply may help recruit scavengers that clear any debris.

We did not detect endothelial cell proliferation until day 8, yet when the tumour burden was low (2–5 × 10^4^ cells implanted to create each tract), ZD4190 prevented outgrowth of most of the implanted cells. This suggests that this agent exerts its effects by blocking vasodilatation and the changes in permeability that aid the process of vessel co-option and increase diffusion of nutrients to the tumour, as well as by modulating endothelial cell proliferation. Dynamic contrast-enhanced magnetic resonance imaging of vascular changes induced by ZD4190 has shown that this agent reduces vessel permeability and modulates blood flow (Checkley *et al*, 2003). An alternative explanation for these findings is that ZD4190 inhibits EGFR signalling, but this receptor is not highly expressed on PDVC57B cells and the drug had no cytotoxic effect *in vitro,* supporting the notion that the primary action is to inhibit angiogenesis mediated by VEGFR2. Some tumour nodules that developed in the ZD4190-treated rodents contained viable malignant cells. Most likely, these did not proliferate as they were too far from blood vessels to obtain adequate nutrients by diffusion. A small number of fibrotic foci also contained proliferating tumour cells, suggesting that they are able to overcome the effects of the drug, or become vascularised by pathways that do not involve VEGFR2.

The treatment effects were different when more than 10^5^ cells were implanted to create each tract. At day 9, the tumour area in the rectus and gastrocnemius muscles was broadly similar for the test and the control groups, although there was a reduction in the MVD of the fibrotic foci in the gastrocnemius. Most likely, the reduction in MVD is confined to tumours in the gastrocnemius as the tumour burden is less and the drug blocks any proangiogenic stimuli. However, this response does not translate into a reduction of tumour area for either muscle at this time, presumably as proliferation of the implanted cells is not critically dependent on the presence of functional new vessels.

When treatment with ZD4190 was continued for 22 days, there was a significant reduction in the tumour area, in the microvascularity and increased necrosis when the test and vehicle-treated groups were compared. This illustrates that as the tumour expands it becomes critically dependent on new vessel formation and the drug modulates the balance of pro- and inhibitory angiogenic factors, mirroring the earlier experience indicating that antiangiogenic agents are more effective when given over a longer term (Ciardiello *et al*, 2003). The difference in the morphology of the ZD4190-treated tumours, with areas of peripheral necrosis, cord-like proliferation of malignant cells and reduced microvascularity can all be attributed to inhibition of VEGFR2 signalling. The infiltrative growth pattern suggests that this change is driven by hypoxia as a consequence of the reduced tissue vascularity. However, blockade of the VEGF pathway is too narrow an approach to prevent outgrowth of residual carcinoma when more than a critical tumour burden is present, as the drug effect is overwhelmed by proangiogenic signals and the tumour may use other compensatory angiogenic pathways.

ZD4190 was replaced by ZD6474 in development, as this agent has better bioavailability (Wedge *et al*, 2002). These agents are highly insoluble and could not be delivered locally, but they can be given orally, and as dosing does not require hospital supervision, patient acceptance is likely to be very good. These antiangiogenic drugs can also be combined successfully with other modalities, including radiotherapy (Williams *et al*, 2004), and we anticipate that blocking multiple targets will come to the fore as the need to provide additional treatment for cases at high risk of local recurrence is recognised. On the basis of the results from this study, it may be inferred that additional treatments should commence as soon after surgery as feasible, as all agents will be most effective when the tumour burden is low. ZD6474 is in phase I clinical trial for unresected stage 3–4 head and neck squamous cell carcinoma in combination with radiation, or with cisplatin and radiation therapy, and should be considered for inclusion in adjuvant treatment regimes for cases with microscopic or molecular evidence of residual carcinoma.

## Figures and Tables

**Table 1:** Effects of ZD4190 on the outgrowth of tumour in a muscle model of residual squamous cell carcinoma after 21 days of administration postoperatively

	**Rectus**						**Gastrocnemius**					
	**2 × 10^4^**			**5 × 10^4^**			**2 × 10^4^**			**5 × 10^4^**		
	**ZD4190**	**C**	**V**	**ZD4190**	**C**	**V**	**ZD4190**	**C**	**V**	**ZD4190**	**C**	**V**
Fibrotic foci no tumour	14	0	0	14	0	0	7	0	0	4	0	0
Fibrotic foci Brdu−tumour	2	0	0	4	0	0	0	0	0	4	0	0
Fibrotic foci Brdu+tumour	3	0	0	4	0	4	1	0	0	2	0	0
Brdu+tumour	0	19	20	0	23	19	0	9	8	0	8	9

Different numbers of PDVC57B cells (2 × 10^4^ and 5 × 10^4^) were implanted to create each tumour tract in the rectus and gastrocnemius muscles and the mice (*n*=5) gavaged daily with ZD4190, vehicle (V) or were untreated (C). The different patterns of tumour outgrowth were assessed by morphological examination and BrdU staining. The number of fibrotic foci forming for each group of rodents and the pattern of Brdu expression is shown. A maximum number of five tracts were implanted into each rectus muscle and two into the gastrocnemius. Significantly more fibrotic foci formed in the rectus muscle (*P*=0.001, *χ*^2^ 38) and the gastrocnemius (*P*=0.001, *χ*^2^ 73.7) when treatments with ZD4190 and the controls or vehicle-treated groups were compared.
